# Human Dystrophin Dp71ab Enhances the Proliferation of Myoblasts Across Species But Not Human Nonmyoblast Cells

**DOI:** 10.3389/fcell.2022.877612

**Published:** 2022-04-25

**Authors:** Manal Farea, Kazuhiro Maeta, Hisahide Nishio, Masafumi Matsuo

**Affiliations:** ^1^ Research Center for Locomotion Biology, Kobe Gakuin University, Kobe, Japan; ^2^ KNC Department of Nucleic Acid Drug Discovery, Faculty of Rehabilitation, Kobe Gakuin University, Kobe, Japan; ^3^ Faculty of Rehabilitation, Kobe Gakuin University, Kobe, Japan

**Keywords:** Dp71ab, Dp71, dystrophin, isoform, DMD, myoblast, cell proliferation

## Abstract

Dystrophin Dp71 is an isoform produced from the Dp71 promoter in intron 62 of the *DMD* gene, mutations in which cause Duchenne muscular dystrophy. Dp71 is involved in various cellular processes and comprises more than 10 isoforms produced by alternative splicing. Dp71ab, in which both exons 71 and 78 are deleted, has a hydrophobic C-terminus that is hydrophilic in Dp71. Therefore, Dp71ab is believed to have different roles from Dp71. Previously, we reported that Dp71ab enhanced the proliferation of human myoblasts. Here, we further characterized Dp71ab, focusing on the activation of cell proliferation. Dp71ab increased the proliferation of immortalized human myoblasts in a dose-dependent manner. In contrast, Dp71 suppressed proliferation in a dose-dependent manner. Consistent with these opposite effects, eGFP-tagged Dp71ab and mCherry-tagged Dp71 showed different cellular distributions, with Dp71ab mostly in the nucleus. Notably, human Dp71ab enhanced the proliferation of rat and mouse myoblasts. Despite these findings, human Dp71ab did not enhance the proliferation of human nonmyoblast cells, including rhabdomyosarcoma cells. We concluded that Dp71ab is a myoblast-specific proliferation enhancer. In further studies, Dp71ab will be employed for the expansion of myoblasts in clinical settings.

## 1 Introduction

The *DMD* gene covers over 2.4 Mb on the X chromosome, is the largest gene in the human genome and produces a 14-kb transcript consisting of 79 exons that encodes dystrophin (Dp427). Mutations in this gene cause Duchenne muscular dystrophy (DMD) (OMIM310200), a fatal progressive muscle wasting disease (Muntoni et al., 2003; Bushby et al., 2010). Dystrophin Dp71 (Dp71), an approximately 71-kDa protein, is the shortest isoform of dystrophin and is produced from the Dp71(G) promoter in intron 62 of the *DMD* gene. Dp71 is encoded by a transcript consisting of the unique exon G1 and *DMD* exons 63–79 (Tadayoni et al., 2012). Dp71 is expressed ubiquitously and plays roles in various cellular processes, including water homeostasis, nuclear architecture, cell adhesion, and cell division and survival (Tadayoni et al., 2012). Dp71 has been mainly studied for its roles in intellectual disability in DMD patients (Naidoo and Anthony, 2020). Recently, Dp71 has attracted increased attention as a factor related to tumorigenesis (Tan et al., 2016a; Tan et al., 2016b; Mauduit et al., 2019).

Dp71 is characterized by multiple types of alternative splicing, resulting in the expression of more than 10 splice variants (Tadayoni et al., 2012; Aragon et al., 2018; Rani et al., 2019). Alternative splicing to skip exons 71 and 78 yields Dp71a and Dp71b, respectively, and simultaneous skipping of exons 71 and 78 produces Dp71ab (Tadayoni et al., 2012). Hence, Dp71ab lacks 13 amino acids encoded in exon 71 and has an elongated C-terminal end due to a frameshift caused by the absence of exon 78 (Tadayoni et al., 2012). The elongated C-terminus of Dp71ab is hydrophobic, in contrast to the hydrophilic C-terminus of Dp71. Based on this structural difference, Dp71ab has been shown to have different functions from Dp71 (Marquez et al., 2003; Herrera-Salazar et al., 2016), but the functional characterization of each isoform has been hampered by the coexpression of isoforms (Aragon et al., 2016; Rani et al., 2019). In our previous study, human Dp71ab was shown to be exclusively expressed in human satellite cells (Farea et al., 2020). Moreover, we demonstrated that Dp71ab enhances myoblast proliferation (Farea et al., 2020). Thus, Dp71ab was shown to be involved in cell proliferation.

For noncurable DMD, many treatment strategies, such as exon skipping and stem cell-based therapy, have been proposed (Matsuo, 1996; Sun et al., 2020b). Subsequently, several antisense oligonucleotides that induce skipping of *DMD* exons have been approved for clinical use by authorities (Schneider and Aartsma Rus, 2021). Satellite cells are muscle stem cells that generate myoblasts by cell division. Myoblasts, in turn, proliferate, differentiate, and fuse to form new myofibers and restore tissue functionality. The regenerative capacity of muscle is diminished in patients with DMD because errors in cell division exhaust the supply of satellite cells (Webster and Blau, 1990; Sacco et al., 2010; Chang et al., 2016). Therefore, myoblast transplantation is one strategy for DMD treatment (Biressi et al., 2020). However, myoblast transplantation therapy has not yet been authorized as a treatment for DMD (Sun et al., 2020a). The clinical application of myoblast transplantation is limited by the lack of a suitable method to expand myogenic cells. To overcome this issue, researchers have studied several distinctive methods (Motohashi et al., 2019). For example, macrophage-derived factors or small molecules have been used to manipulate the signaling pathways involved in myogenic cell proliferation (Judson et al., 2018; Wehling Henricks et al., 2018; Li et al., 2021). Recently, amino acids were shown to enhance muscle stem cell proliferation (Lin et al., 2020; Gheller et al., 2021). Although glycine attenuates muscle wasting (Ham et al., 2014; Ham et al., 2019), this molecule has never been applied for DMD treatment to ameliorate muscle wasting. However, glutamine supplementation was conducted in DMD patients, resulting in inhibition of whole-body protein degradation (Mok et al., 2006). Unfortunately, glutamine supplementation did not show functional benefits in a randomized crossover trial (Mok et al., 2009).

The application of myoblast transplantation therapy has been expanding to treat various disease involving muscle atrophy, such as muscular dystrophies and cardiac failure (Partridge et al., 1998; Menasche et al., 2001; Périé et al., 2014). Establishment of a method to expand myoblast proliferation in a myoblast-specific manner is urgently needed for myoblast transplantation therapy. Here, human dystrophin Dp71ab was further characterized with regard to its effect on cell proliferation. Dp71ab enhanced the proliferation of human myoblasts in a dose-dependent manner. Notably, this molecule enhanced the proliferation of mouse and rat myoblasts. Although Dp71 was shown to enhance myoblast proliferation across species, it did not enhance the proliferation of human nonmyoblast cells. Dp71ab was identified as a myoblast-specific proliferation enhancer. Thus, Dp71ab can be employed for the expansion of myoblasts in clinical settings.

## 2 Materials and Methods

### 2.1 Cells

Human myoblasts generated by the immortalization of primary cultured human myogenic cells were a kind gift from Dr. Hashimoto (Hashimoto et al., 2006). CRL-2061 and CCL-136 rhabdomyosarcoma cells, HeLa cervical carcinoma cells, SH-SY5Y neuroblastoma cells, HepG2 hepatoma cells, AGS gastric adenocarcinoma and HEK human embryonal kidney cells, and rat L6 cells and mouse C2C12 myoblasts were obtained from the American Type Culture Collection (ATCC; Manassas, VA, United States). Cells were cultured in Dulbecco’s modified Eagle’s medium (DMEM; Gibco Life Technologies, Waltham, MA, United States), except for CRL-2061 that were cultured in RPMI (Gibco Life Technologies, Waltham, MA, United States) and SH-SY5Y and HepG2 that were cultured in Minimum Essential Medium (MEM) (Gibco by Life Technologies, Grand Island, NY, United States). Media were supplemented with 10% fetal bovine serum (FBS; Gibco Life Technologies) and 1% antibiotic-antimycotic solution (AA; Gibco Life Technologies) at 37°C in a 5% CO_2_ humidified incubator.

### 2.2 Myoblast Differentiation

Myoblasts (1 × 10^5^ cells/well) were plated in 12-well plates covered by collagen-coated coverslips and cultured in DMEM. After 24 h, the growth medium (DMEM) was replaced by two types of differentiation medium: TIS medium consisting of DMEM with bovine holo-transferrin (5 μg/ml), porcine insulin (10 μg/ml), sodium selenite (10 nM), 2% FBS and 1% AA and HS medium consisting of DMEM with 2% horse serum. C2C12 cells were used as a control in the current study. For differentiation of immortalized human myogenic cells, two differentiation media, TIS and IHS, were used as it was unknown which medium is suitable for myoblast differentiation.

After 10 days of culture, the cells were examined for the expression of myosin heavy chain protein and myogenic genes. For immune staining, the cells were rinsed with Dulbecco’s PBS (DPBS), fixed with 4% paraformaldehyde for 15 min, permeabilized with 0.2% TX-100 (TX-100) (T8787,Sigma-Aldrich Co., St Louis, MO, United States) for 10 min and incubated with blocking buffer (5% BSA in DPBS). The cells were then incubated overnight with 0.3 μg/ml anti-myosin heavy chain antibody (ab124205, Abcam, Cambridge, United Kingdom), followed by Alexa Fluor secondary antibody (goat anti-rabbit antibody, ab150077, Abcam). The cells were stained with NucBlue Live Ready Probes Reagent (Hoechst 33342, Thermo Fisher Scientific) for 20 min, and coverslips were mounted with Fluoro-Keeper Antifade reagent (Nacalai Tesque, Inc., Kyoto, Japan) to prevent fading. Cells in a microscopic field of each well were captured by the 20 x Plan Fluor lens of a BZ-X710 fluorescence microscope (Keyence, Osaka, Japan). The fusion index (%) was calculated for each medium by dividing the number of nuclei within multinucleated myofibers by the total number of nuclei. For mRNA analysis, total RNA was extracted from cultured cells using a High Pure RNA Isolation kit (11828665001, Roche). Total RNA from skeletal muscle was obtained from Clontech Laboratories, Inc., (Mountain View, CA, United States, 636534) and cDNA was synthesized from 0.5 μg of total RNA using murine reverse-transcriptase (28025-021, Thermo Fisher Scientific, Carlsbad, CA) and random primers (58875, Life Technologies Corp.) as described before (Farea et al., 2020) . The expression of paired box transcription factor 3 and 7 (*PAX3* and *PAX7*, respectively) and myogenic regulatory factor (*Myf5*, *MyoD*, and *myogenin*) mRNAs was examined by RT-PCR amplification using sets of primers for *PAX3* (forward,: 5′-GTC AAC​CAG​CTC​GGC​GGT​GTT​T-3′; reverse, 5′-ATG​GCA​CCA​GGA​CGT​ATG​GT-3′); *PAX7* (forward, 5′-GGG​TCT​TCA​TCA​ATG​GGC​GA-3′; reverse, 5′-GTC​ACA​GTG​CCC​ATC​CTT​CA-3′); *Myf* (forward, 5′-TGG​ATG​GCT​GCC​AGT​TCT​CA-3′; reverse, 5′-CCGATCCA GGTGGTGGACT-3′); *MyoD* (forward, 5′-AGC​ACT​ACA​GCG​GCG​ACT -3′; reverse, 5′-CGA​CTC​AGA​AGG​CAC​GTC -3′); and *myogenin* (forward, 5′-CTG​CTC​AGC​TCC​CTC​AAC​CA-3′; reverse, 5′GGT​CAG​CCG​TGA​GCA​GAT​GAT-3′). GAPDH mRNA was also amplified using a set of primers (forward, 5′-CCC​TTC​ATT​GAC​CTC​AAC-3'; reverse, 5′-TTC​ACA​CCC​ATG​ACG​AAC-3′) and used as a control as described previously (Farea et al., 2020). PCR-amplified products were electrophoresed using DNA 1000 LabChip kits on an Agilent 2100 Bioanalyzer (Agilent Technologies, Santa Clara, CA, United States).

### 2.3 Dp71ab, eGFP-Tagged Dp71ab, Dp71, and mCherry-Tagged Dp71 Plasmids

Plasmids encoding Dp71ab, Dp71, and their derivatives tagged with eGFP and mCherry at N-terminus, respectively, were constructed by inserting the coding sequences of the respective sequences into the mammalian expression vector pcDNA3 with a CMV promoter (Invitrogen, Thermo Fisher Scientific, Inc.). DNA containing the respective sequences was synthesized by FASMAC Co., Ltd., as described previously (Farea et al., 2020).

### 2.4 Cell Proliferation Assay

Myoblasts (1 × 10^4^ cells/well) were plated in triplicate in 96-well plates. After 24 h, the cells were transfected with Dp71, Dp71ab and mock plasmids dissolved in 0.3 μl of Lipofectamine 3000 (Thermo Fischer Scientific, Carlsbad, CA) according to the manufacturer’s protocol. Three hours later, the media were replaced with DMEM. At 24, 48 and 72 h after transfection, 10 μl of CCK-8 solution (Dojindo Laboratories, Kumamoto, Japan) was added to each well, and the absorbance of each well at 450 nm was determined using a microplate reader (ARVO X3, Perkin Elmer, MA, United States). Cell proliferation was also analyzed by counting cells under a microscope after Hoechst 33342 staining. Cells in a 1 mm^2^ area of each well captured by the 4x Plan Fluor lens of a BZ-X710 fluorescence microscope (Keyence Corp., Osaka, Japan) were counted at 0, 24, 48, and 72 h after transfection and analyzed using BZ-X analytic software (Keyence Corp.). The same area of each well was analyzed at each time point. The numbers of cells is shown as the average of three wells containing the same cell populations. The results shown are representative of three identical experiments. Glycine (12997-42, Nacalai Tesque, Kyoto, Japan), glutamine (16948-04, Nacalai Tesque), alanine (12998-32, Nacalai Tesque) and alpha-ketoglutarate (K1128, Sigma-Aldrich Co., St Louis, MO, United States) were added to the culture medium at a concentration of 1 mM as reported before.

### 2.5 Localization of Dp71 and Dp71ab in Myoblasts

Myoblasts (1 × 10^3^ cells/well) were plated in 96-well plates. After 24 h, the cells were transfected with 10 ng of the eGFP-tagged Dp71ab or mCherry-tagged Dp71 plasmids in 0.1 μl of Lipofectamine 3000 (Thermo Fischer Scientific) according to the manufacturer’s protocol. Three hours later, the medium was replaced with FluoroBrite DMEM medium (A18967-01, Gibco Life Technologies) supplemented with Hoechst 33342. Cells in a microscopic field of each well were captured by the 20 × Plan Fluor lens of a BZ-X710 fluorescence microscope (Keyence Corp., Osaka, Japan) at 24, 48, and 72 h after transfection and analyzed using a BZ-X 800 analyzer (Keyence Corp ). The cellular localization of each type of fluorescence was classified into three patterns: cytoplasm, nucleus and nucleus/cytoplasm. The cell number for each pattern was counted, and the percentage of each pattern in approximately 400 cells was calculated.

### 2.6 Statistical Analyses

All assays were repeated 3 times to ensure reproducibility. The results reported as the mean ± SE were analyzed by one-way ANOVA and LSD. The results reported as the mean ± SD were analyzed by ANOVA for comparisons of three or more groups and Student’s t tests for comparisons between two groups. All statistical analyses were performed using SPSS software (version 17.0; SPSS, Inc., Chicago, IL, United States), with *p* < 0.05 considered statistically significant.

## 3 Results

### 3.1 Enhanced Proliferation of Human Myoblasts by Dp71ab

#### 3.1.1 Characterization of Human Myoblasts Used in This Study

**FIGURE 1:**
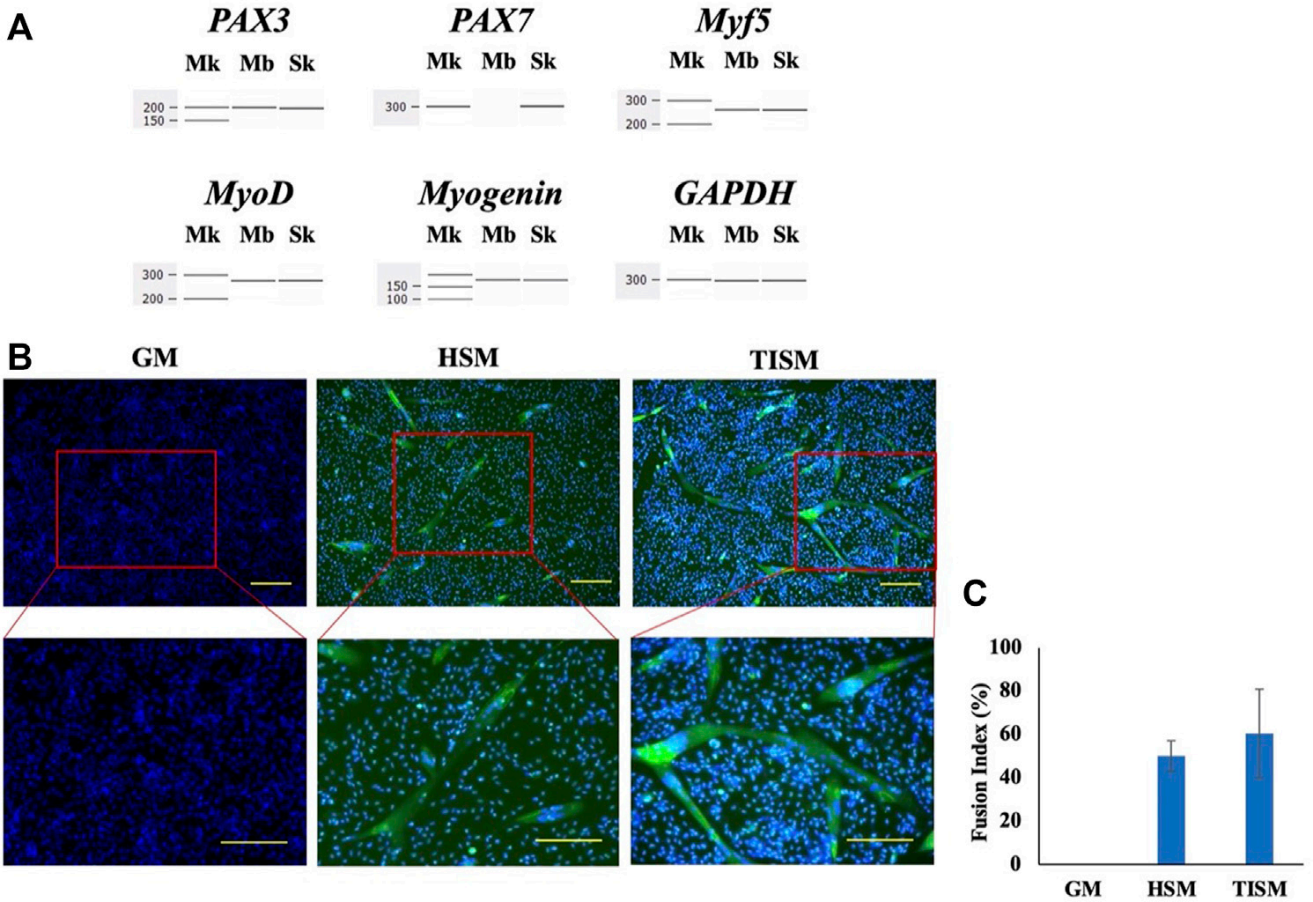
Characterization of human immortalied myoblasts. **(A)**. Expression of myogenic genes. The mRNA levels of the *PAX3, PAX7, Myf5, MyoD*, and *myogenin* genes in human immortalized myoblasts were assessed by RT-PCR using *GAPDH* mRNA as a control. Electropherograms of the amplified products are shown with size markers on the left (Mk). All amplifications except that of *PAX7* resulted in a band at the expected size (Mb) as amplified products from skeletal muscles (Sk). **(B)**. Differentiation of human immortalized myoblasts. Human immortalized myoblasts were cultured in two types of differentiation (HSM and TISM) and one type of growth (GM) media for 10 days and analyzed. Merged results of immunostaining of myosin heavy chain and Hoechst 33342 staining are shown. In differentiated cells, spindle-shaped and multinuclear cells were observed microscopically (HSM, TISM), whereas they were not observed in the cells cultured in growth medium (GM). Myosin heavy chain signals were observed as green signals in the cells cultured in differentiation medium (TISM, HSM) but not in the cells in growth medium (GM). Squares marked by red lines in the upper panels were enlarged and are shown in the lower panels. Scale bars = 100 μm. **(C)**. The fusion index. The fusion index (%) was calculated for each medium and is shown as bars.

In human myoblast studies, we used immortalized human myoblasts established by Dr. Hashimoto (Hashimoto et al., 2006). To characterize stored immortalized myoblasts, we determined the mRNA expression of the *PAX3, PAX7, Myf5, MyoD*, and myogenin genes by RT-PCR and amplification. We obtained products of the expected size of all transcripts except *PAX7* (Figure 1A), indicating PAX3(+), PAX7(-), Myf5(+), MyoD(+), and myogenin(+) cells. This finding was consistent with the characteristics of myoblasts (Brusa et al., 2020). Next, the differentiation of myoblasts was examined by culturing them in differentiation medium. After 10 days of culture, spindle-shaped and multinuclear cells were microscopically identified (Figure 1B). Furthermore, myosin heavy chain staining revealed positive staining in the cultured cells (Figure 1B). The fusion index was high in cell cultured in differentiation medium (Figure 1C). These results confirmed that human immortalized myoblasts maintain the ability to differentiate into myotubes even after long-term storage.

#### 3.1.2 Enhanced Proliferation of Human Myoblasts by Dp71ab

**FIGURE 2:**
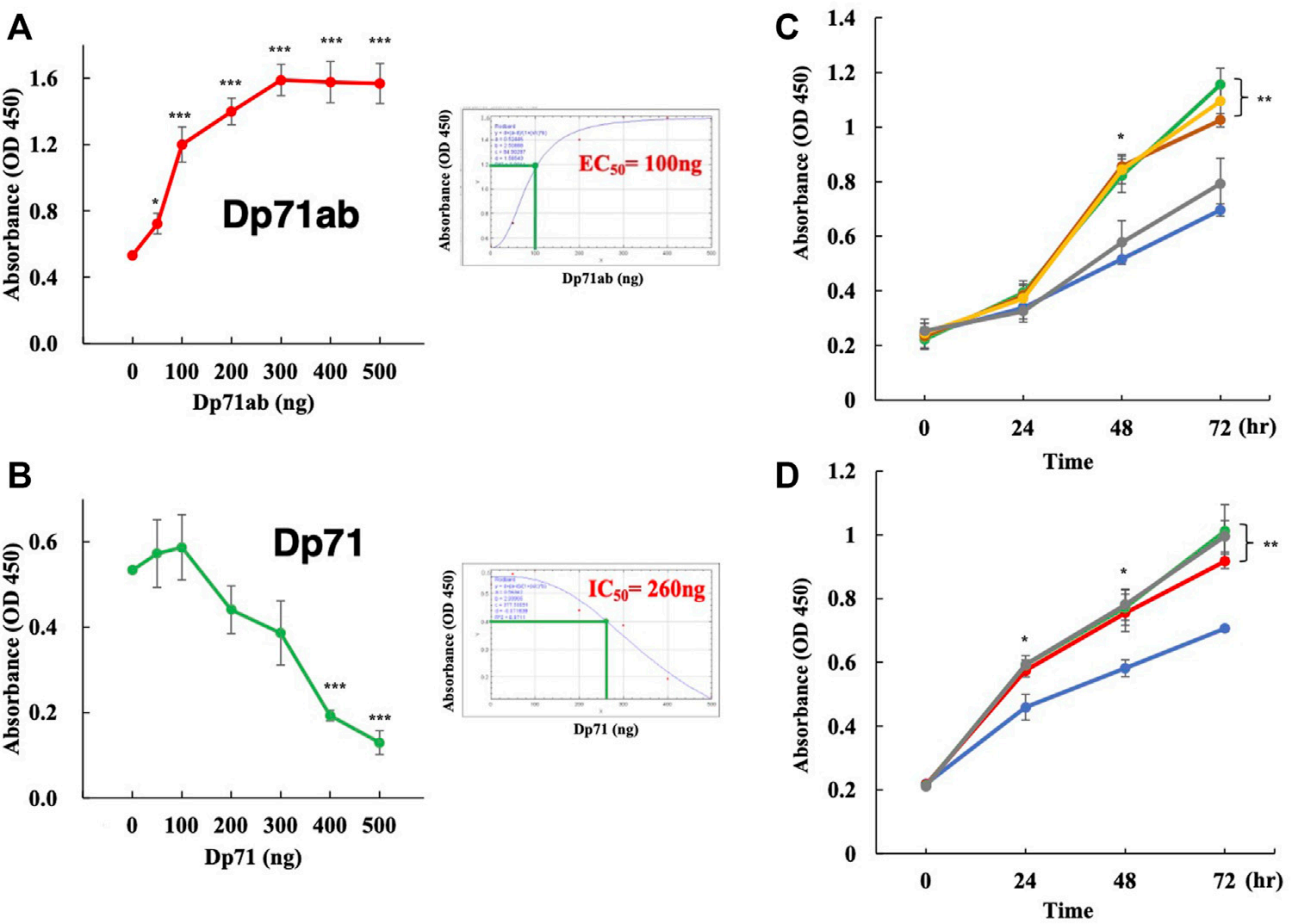
Dp71ab, but not Dp71, enhances the proliferation of myoblasts. Myoblasts were transfected with Dp71ab and Dp71 plasmids at different doses and cultured for 72 h. Cell proliferation was assessed by the CCK-8 assay. The absorbance was determined in each well. Data are expressed as the mean ± SEM of three independent experiments. The absorbance increased linearly at doses from 0 to 300 ng of the Dp71ab plasmid **(A)** left. The EC_50_ was calculated as 100 ng **(A)** right. In contrast, Dp71 decreased the absorbance linearly from 100 to 500 ng of the plasmid **(B)** left. The IC_50_ was calculated as 260 ng **(B)** right. Glycine, glutamine, alanine and alpha-ketoglutarate were examined for their effect on myoblast proliferation. The addition of glycine (green), glutamine (brown) and alanine (yellow), but not alpha-ketoglutarate (gray), in the culture medium increased the absorbance significantly compared with that of the nontreated cells (blue) (*p* < 0.05 and *p* < 0.001 after 48 and 72 h, respectively) **(C)**. The addition of glycine to the culture medium of the Dp71ab-transfected myoblasts (gray) did not increase the absorbance compared to those in the Dp71ab-transfected (red) and glycine-supplemented (green) cells. However, all treatments increased the absorbance significantly compared to that of the nontreated cells (blue) **(D)**. * = *p* < 0.05, ** = *p* < 0.01, *** = *p* < 0.001.

The Dp71ab-mediated enhancement of myoblast proliferation was analyzed by transfecting different amounts of the Dp71ab plasmid into human myoblasts. Cell proliferation was assessed by CCK-8 assays of cells incubated for 72 h. As expected, the absorbance linearly increased from 0.5 to 1.6 by increasing the dose of the plasmid from 0 to 300 ng and plateaued at 400 and 500 ng (Figure 2A). Based on these results, the half maximal effective concentration (EC_50_) was determined to be 100 ng. However, transfection of the Dp71 plasmid at 0, 50 and 100 ng did not substantially change the absorbance (Figure 2B). Notably, 200 ng of the Dp71 plasmid decreased the absorbance, and thereafter, the absorbance decreased in a dose-dependent manner to 500 ng of the Dp71 plasmid. The half maximal inhibitory concentration (IC_50_) was determined to be 260 ng (Figure 2B). We found that Dp71ab increased the absorbance to nearly 3 times higher than that of the nontreated cells, and Dp71 decreased it to less than 10% of the control value. These results indicated that Dp71ab and Dp71 have opposite effects on myoblast proliferation.

#### 3.1.3 Effects of Amino Acids on Myoblast Proliferation

Glycine, glutamine, alanine and alpha-ketoglutarate have been reported to enhance proliferation of muscle stem cells (Lin et al., 2020; Shang et al., 2020; Li et al., 2021). Thus, their effects on the proliferation of human myoblasts were examined. The addition of glycine, glutamine and alanine, but not alpha-ketoglutarate, to the culture medium of human myoblasts significantly increased the absorbance determined by CCK-8 assays compared with that of the untreated cells at 72 h of incubation (Figure 2C). Since glycine is a simple amino acid and has been well studied in muscle cell proliferation (Ham et al., 2019; Lin et al., 2020; Gheller et al., 2021), we examined the additional effect of this amino acid on the proliferation of Dp71ab-transfected myoblasts. However, an additional increase in absorbance was not observed in the myoblasts transfected with the Dp71ab plasmid and cultured in glycine-supplemented medium (Figure 2D). This finding indicated no additional enhancement of myoblast proliferation by the combination of Dp71ab and glycine. Dp71ab may saturate the proliferative capacity of myoblasts.

### 3.2 Nuclear Localization of Dp71ab

**FIGURE 3:**
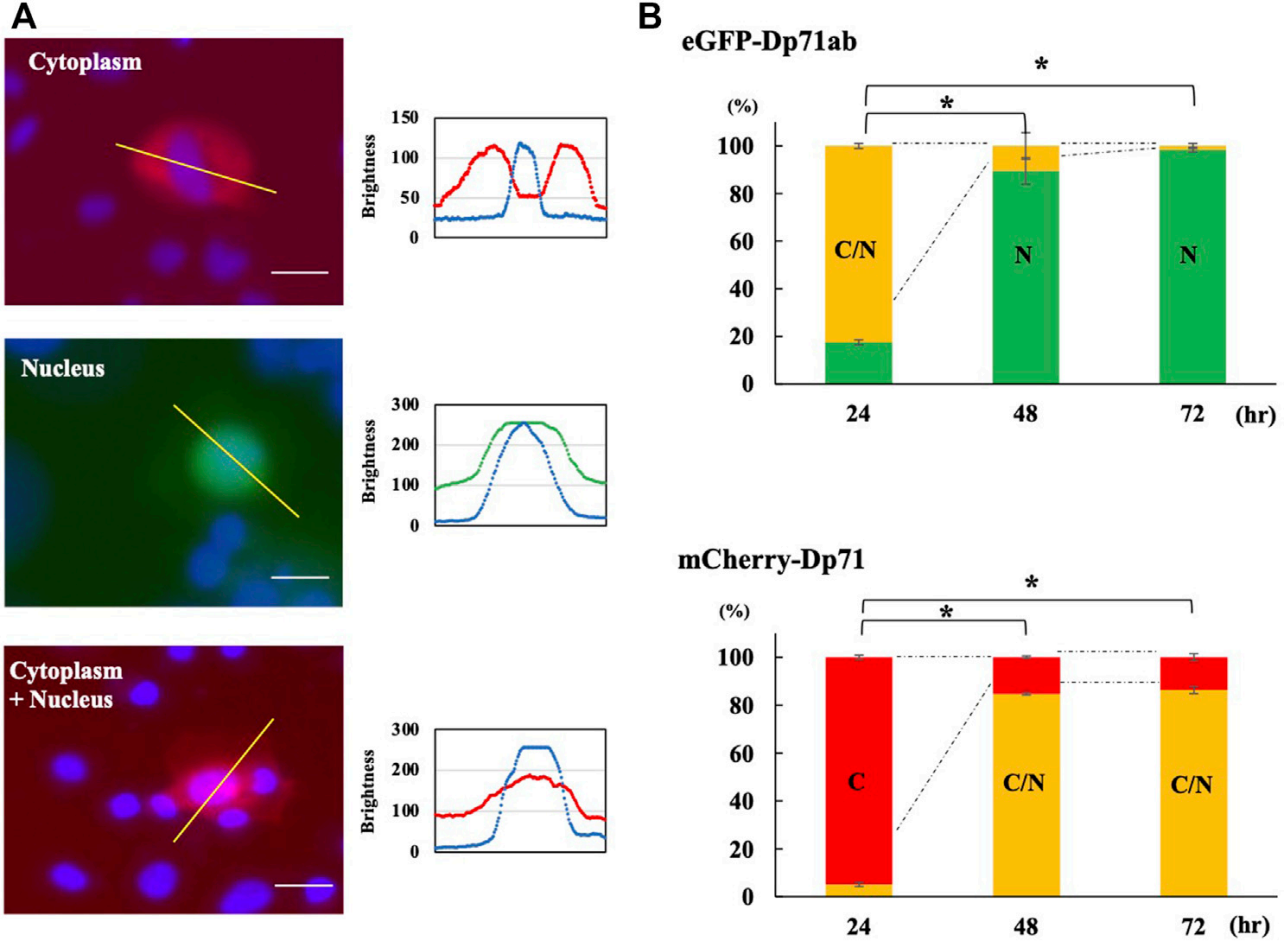
Cellular localization of Dp71ab and Dp71 in myoblasts. eGFP-tagged Dp71ab and mCherry-tagged Dp71 were expressed in myoblasts, and fluorescence images of red mCherry-Dp71 and green eGFP-Dp71a were obtained at 24, 48 and 72 h. The cellular localization of the expressed proteins was determined by visualizing the nucleus with Hoechst 33342 staining and classified into three groups: cytoplasm (C), cytoplasm + nucleus (cytoplasm/nucleus) (C/N) and nucleus (N). Representative images of the three groups of cellular localization (left of each group) and sagittal section (right of each group) results are shown **(A)**. In the cytoplasmic group, mCherry-Dp71 was present in the cytoplasm, leaving the nucleus open (left). The distribution of signals along the bar in the top figure is shown (bottom). A red signal was present, avoiding the nucleus (blue signal). In the nuclear group, green eGFP-tagged Dp71ab was present in the nucleus (middle). In the cytoplasm/nucleus group, mCherry-Dp71 was present in both the cytosol and nucleus (right). The percentages of cells classified into the three groups were calculated at 24, 48 and 72 h and are shown as bars **(B)**. At 24 h, 94.9% of the myoblasts transfected with Dp71 belonged to the cytoplasm group. In contrast, 82.5% of the myoblasts transfected with Dp71ab belonged to the cytoplasm/nucleus group. At 72 h, 86.3% of the myoblasts transfected with Dp71 belonged to the cytoplasm/nucleus group. In contrast, 98.4% of the myoblasts transfected with Dp71ab belonged to the nucleus group. The percentage of the major group at 24 h showed significant changes at 48 and 72 h * = *p* < 0.001 compared with the major group at 24 h.

Since Dp71ab and Dp71 showed opposite effects on myoblast proliferation, we hypothesized that these proteins are differentially localized in myoblasts. Therefore, plasmids encoding eGFP-tagged Dp71ab and mCherry-tagged Dp71 were transfected into myoblasts, and their fluorescence was analyzed by fluorescence microscopy at 24, 48 and 72 h of culture. The pattern of cell localization of fluorescence was classified into three groups: the cytoplasm, nucleus and nucleus/cytoplasm groups (Figure 3A). The percentages of cells classified in the three groups were calculated, and their changes were analyzed (Figure 3B). At 24 h of incubation, 94.9% of the mCherry-Dp71-positive cells were classified in the cytoplasm group, corresponding to the site of protein synthesis in the cytoplasm. In contrast, 82.5 % of the eGFP-Dp71ab-positive cells belonged to the nucleus/cytoplasm group, and the rest belonged to the nuclear group, indicating the nuclear preference of Dp71ab. Thereafter, the cellular distribution of eGFP-Dp71ab and mCherry-Dp71 followed a different fate. At 72 h, 98.4% of the eGFP-Dp71ab-positive cells were classified in the nucleus group. In contrast, 86.3% of the mCherry-Dp71-positive cells were classified in the nucleus/cytoplasm group. These results revealed a clear difference in cellular localization between Dp71ab and Dp71, indicating that Dp71ab is a nuclear protein.

### 3.3 Enhancement of L6 Rat and C2C12 Mouse Myoblast Proliferation by Human Dp71ab

**FIGURE 4:**
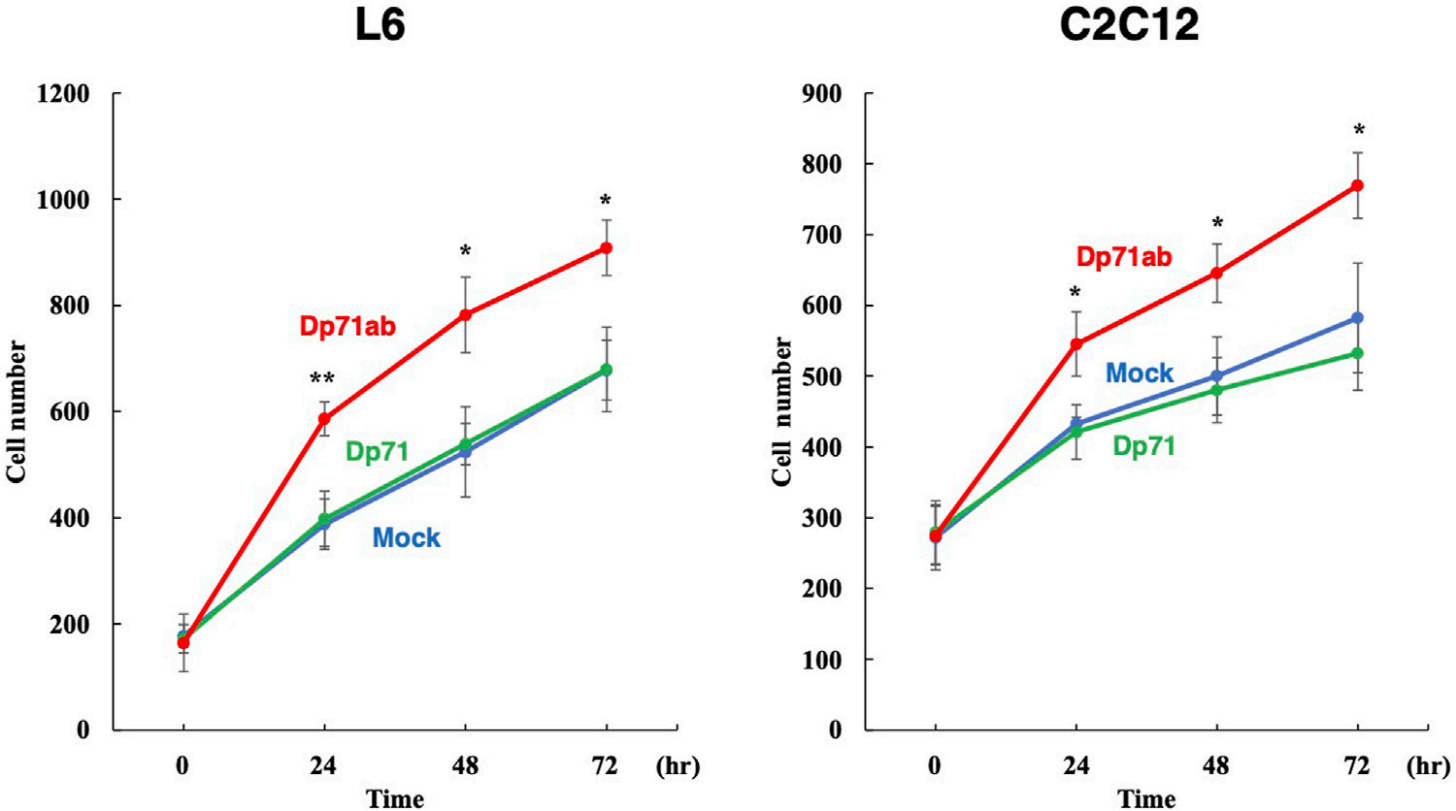
Enhancement of L6 rat and C2C12 mouse myoblast proliferation by Dp71ab. L6 rat and C2C12 mouse myoblasts were transfected with Dp71ab, Dp71 and mock plasmids and incubated for 72 h. As expected, Dp71ab (red) significantly increased the number of L6 and C2C12 cells compared to that of the Dp71 (green) or mock (blue) transfected cells (134 and 132%, respectively). Human Dp71ab enhanced the proliferation of myoblasts across species. * = *p* < 0.05, ** = *p* < 0.001.

We investigated whether human Dp71ab can enhance the proliferation of myoblasts in rats and mice. The amino acid sequences of rat and mouse Dp71ab differ from those of humans by three and two amino acid residues, respectively. L6 rat and C2C12 mouse myoblasts have been frequently employed in studies of muscle stem cell biology (Ilarraza-Lomeli et al., 2007; Niioka et al., 2018). These cell lines were transfected with the human Dp71ab plasmid, and their cell number was analyzed microscopically. As expected, after Dp71ab treatment (red), the cell numbers of both C2C12 and L6 cells were significantly higher than that of the untransfected cells (mock) at 24, 48 and 72 h of the culture. At 72 h, the increase was calculated to be 132% (*p* < 0.05) and 134% (*p* < 0.05) of that of the nontransfected C2C12 and L6 cells, respectively (Figure 4) (Supplementary Figure S1). The Dp71-transfected cells (green) did not show a significantly higher cell number than the nontreated cells (blue). Human Dp71ab was shown to be an enhancer of myoblast proliferation across species.

### 3.4 No Enhancement of Proliferation of Human Nonmyoblast Cells by Dp71ab

**FIGURE 5:**
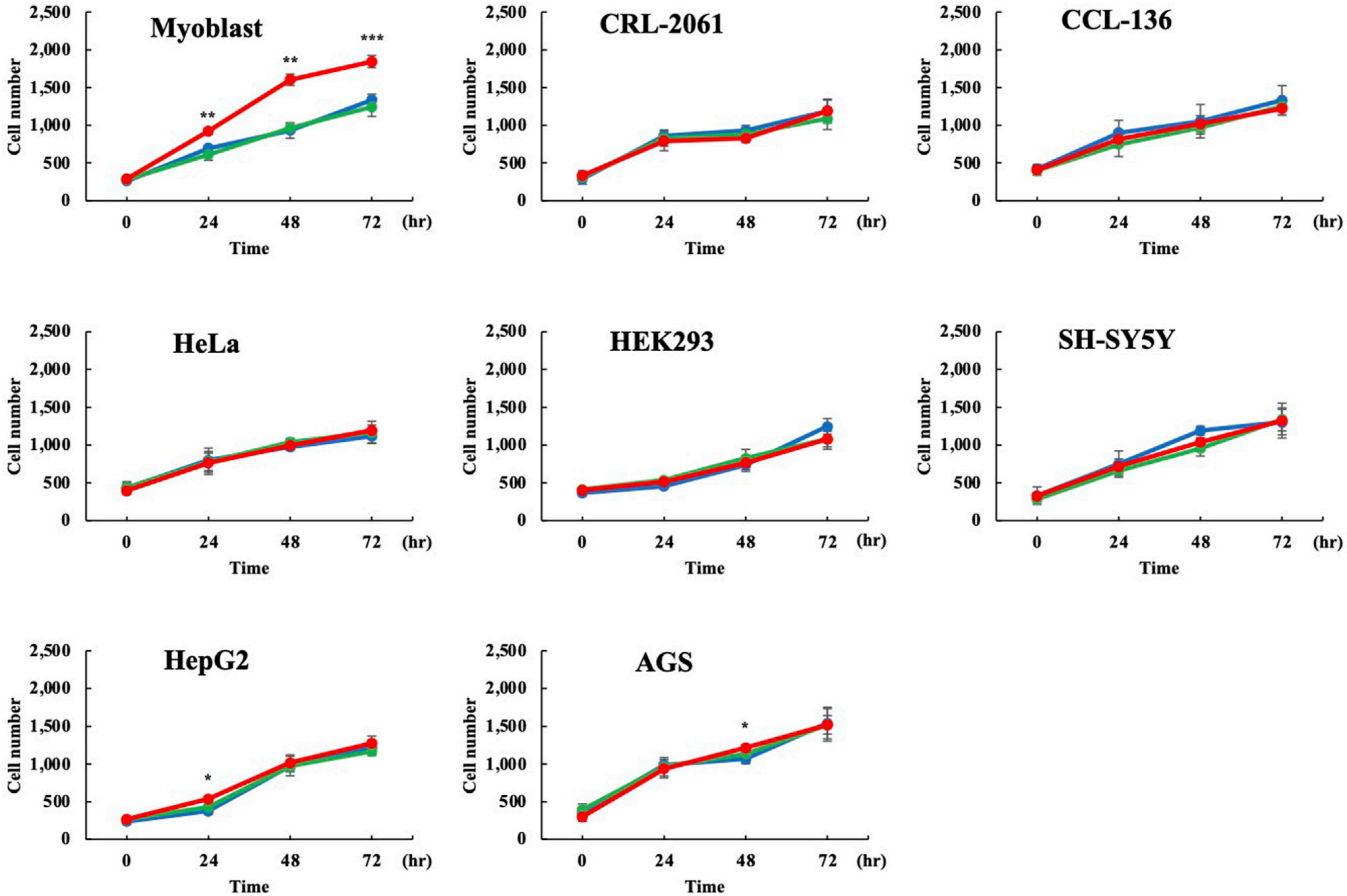
No enhancement of proliferation of human nonmyoblast cells by Dp71ab. Dp71ab, Dp71 and mock vectors were transfected into eight human cell lines, and their cell numbers were assessed microscopically at 0, 24, 48 and 72 h (red, green, and blue lines, respectively). Dp71 did not increase the cell number in all cell lines examined compared with the control (myoblast, CRL-2061, CCL-126, HeLa, HEK293, SH-SY5Y, HepG2 and AGS). As expected, Dp71ab significantly increased the number of myoblasts compared with that of the mock plasmid-transfected cells (*p* < 0.001). However, Dp71ab had no effect in the other seven cell lines, including two myogenic cell lines (CRL-2061 and CCL-136). * = *p* < 0.05, ** = *p* < 0.01, *** = *p* ≤ 0.001.

Human Dp71ab is believed to enhance the proliferation of human cells other than myoblasts. Thus, Dp71ab was examined for its ability to enhance the proliferation of human cells. Dp71ab was overexpressed in seven human cell lines by transfecting the Dp71ab plasmid using Dp71 and mock plasmids as a control. Proliferation was analyzed by a cell counting assay at 0, 24, 48 and 72 h (Figure 5) (Supplementary Figure S2). Unexpectedly, Dp71ab did not enhance the proliferation of any of the seven human cell lines at the end of our analysis (CRL-2061 and CCL-136 rhabdomyosarcoma cells, HeLa cervical carcinoma cells, HEK293 human embryonal cells, SH-SY5Y neuroblastoma cells, HepG2 hepatoma cells, and AGS gastric cancer cells) (Figure 5). Notably, Dp71ab did not enhance the proliferation of the two rhabdomyosarcoma cell lines. However, Dp71 neither enhanced nor suppressed the proliferation of all examined cells. We concluded that Dp71ab is a myoblast-specific proliferation enhancer.

## 4 Discussion

The role of human dystrophin Dp71ab in cell proliferation was characterized and compared with that of Dp71. The following results were obtained: 1) Dp71ab enhanced the proliferation of human myoblasts, while Dp71 at high concentrations suppressed proliferation. 2) Dp71ab was localized in the nucleus, while Dp71 was distributed diffusely in myoblasts. 3) Dp71ab enhanced myoblast proliferation, which was not additionally enhanced by glycine. 4) Dp71ab enhanced the proliferation of rat and mouse myoblasts but not human nonmyoblast cells. We found that Dp71ab and Dp71 exerted opposite effects on myoblast proliferation, although they are related isoforms differing only in exon 71 and 78 content. Dp71ab is a myoblast proliferation enhancer across species but not a cell proliferation enhancer of nonmyoblast human cells. Dp71ab was shown to specifically enhance the proliferation of myoblasts.

Dp71ab is shorter than Dp71 at the mRNA level due to the deletion of exons 71 and 78. At the protein level, however, Dp71ab is larger than Dp71, producing 31 unique amino acids in the C-terminal end instead of the 13 amino acids of Dp71 because of a frameshift due to deletion of exon 78. As a result, Dp71ab consists of 622 amino acids, seven amino acids longer than Dp71. Dp71ab is characterized by a hydrophobic C-terminal that has been identified in Dp427 expressed in myotonic dystrophy patients and recognized as the fetal form of Dp427 (Rau et al., 2015). However, no further studies on this hydrophobic C-terminal of Dp427 have been performed. In this study, Dp71ab with a hydrophobic C-terminal was localized to the nucleus, whereas Dp71 was diffusely localized in both the cytosol and the nucleus of myoblasts. These results suggested that the hydrophobic C-terminal sequence of Dp71ab is key for nuclear localization.

As Dp71ab is a minor isoform of Dp71, which comprises more than 10 isoforms (Rani et al., 2019), studies on human Dp71ab are limited. To date, Dp71ab has been identified in human cells such as cultured amniocytes (Austin et al., 1995), embryonal kidney cells (Nishida et al., 2016), and satellite cells (Farea et al., 2020), but no functional study has been conducted. In rats, Dp71ab was shown to form the Dp71ab/dystroglycan complex in the nucleus of pheochromocytoma-derived PC12 cells (Villarreal-Silva et al., 2010; Aragon et al., 2016). During nerve growth factor-induced differentiation of PC12 cells, the expression of Dp71ab increased (Marquez et al., 2003), and the composition of the Dp71ab/dystroglycan complex changed (Romo-Yanez et al., 2007). Nevertheless, Dp71ab has never been studied for its physiological roles, even in rats. We elucidated the roles of Dp71ab in cell proliferation by identifying its localization in the nucleus for the first time.

Dp71 is known to form a transmembrane complex (so-called dystrophin dystroglycan complex, DAG complex) through binding of its C-terminal domain with β-dystroglycan expressed in both plasma membranes and the nuclear envelope (Bozzi et al., 2009). The diffuse cellular localization of Dp71 disclosed in this study was consistent with the expression patterns of β-dystroglycan. Furthermore, the diffuse distribution of Dp71 was consistent with a shuttle movement of Dp71 between the nucleus and the cytoplasm (Suarez-Sanchez et al., 2014). Since the ZZ finger motifs encoded by exons 68 and 69 of the *DMD* gene are a nuclear targeting sequence (Suarez-Sanchez et al., 2014; Rodriguez-Munoz et al., 2015), the identification of both Dp71 and Dp71ab in the nucleus is logical. The difference in cellular localization between Dp71ab and Dp71 is believed to be due to the difference in the C-terminal ends of these isoforms. The hydrophobic C-terminal end of Dp71ab may bind unidentified nuclear proteins that trigger enhancement of myoblast proliferation. Further study is needed to confirm this hypothesis.

Notably, human Dp71ab enhanced the proliferation of rat L6 and mouse C2C12 myoblasts, regardless of a small amino acid sequence difference in Dp71ab. In contrast, Dp71 neither enhanced nor suppressed proliferation of rat and mouse myoblasts. This functional difference between Dp71ab and Dp71 was supposed due to the cellular localization difference of these proteins that harbor different C-terminus. Our results clearly indicated that human Dp71ab can promote proliferation in nonhuman cells. Although Dp71ab is believed to be a universal cell proliferation enhancer, it did not enhance the proliferation of human nonmyoblast cells, including rhabdomyosarcoma cells. This result clearly indicated the myoblast-specific activity of human Dp71ab. However, the cell-specific mechanism of Dp71ab is still unclear. Identification of the cell-specific activity of Dp71ab was surprising because Dp71ab is a minor isoform of the ubiquitously expressed Dp71 (Tadayoni et al., 2012; Nishida et al., 2016; Rani et al., 2019). Nevertheless, in our previous study, we found that only Dp71ab was expressed in satellite cells (Farea et al., 2020). This result indicated that Dp71ab, but not full-length Dp71, is an important isoform in satellite cells.

Myoblast transplantation therapy has been studied for the treatment of not only muscular dystrophies but also cardiomyopathies. Clinically, this therapy has been tested for three conditions. For DMD treatment, heterologous myoblasts were transplanted to deliver the normal *DMD* gene (Biressi et al., 2020). For oculopharyngeal muscular dystrophy patients, autologous myoblasts were transplanted into affected tissues from healthy parts of the body (Périé et al., 2014). Recently, autologous myoblast cell sheets were transplanted for end-stage ischemic cardiomyopathy, improving the 5-year survival rate (Kainuma et al., 2021). Overall, the generation of sufficient numbers of transplantable myoblasts remains a major obstacle to myoblast therapy. Therefore, many methods to expand myoblasts, including amino acids, have been studied. Recently, glutamine, glycine and alpha-ketoglutarate have been reported to enhance muscle stem cell proliferation (Lin et al., 2020; Zielińska Górska et al., 2020; Li et al., 2021). These compounds were confirmed to enhance human immortalized myoblasts in this study. However, no additive enhancement by the combination of Dp71ab and glycine was observed. Establishment of a suitable myoblast expansion method is urgently needed in the myoblast transplantation therapy field (Motohashi et al., 2019). Since Dp71ab enhanced myoblast proliferation in a myoblast-specific manner, we believe that Dp71ab can generate a high yield of stem cells for myoblast transplantation therapy. Therefore, Dp71ab with myoblast specificity may be a suitable molecule to expand myoblasts. This issue should be examined further in clinical models.

## 5 Limitations

The exact mechanisms of Dp71ab in myoblast proliferation are unclear. In particular, it is unknown how Dp71ab exerts its cell specificity. Therefore, identification of the binding proteins of Dp71ab in the nucleus of myoblasts should be performed in the future.

## Data Availability

The raw data supporting the conclusions of this article will be made available by the authors, without undue reservation.
