# Supplementary material for: Human Dystrophin Dp71ab Enhances the Proliferation of Myoblasts Across Species But Not Human Nonmyoblast Cells
Source: Front Cell Dev Biol. 2022 Apr 25;10:877612. doi: 10.3389/fcell.2022.877612 (PMC9081641; doi:10.3389/fcell.2022.877612)
Supplement: Supplementary file 1 [file Presentation1.pdf]

Supplementary Figures

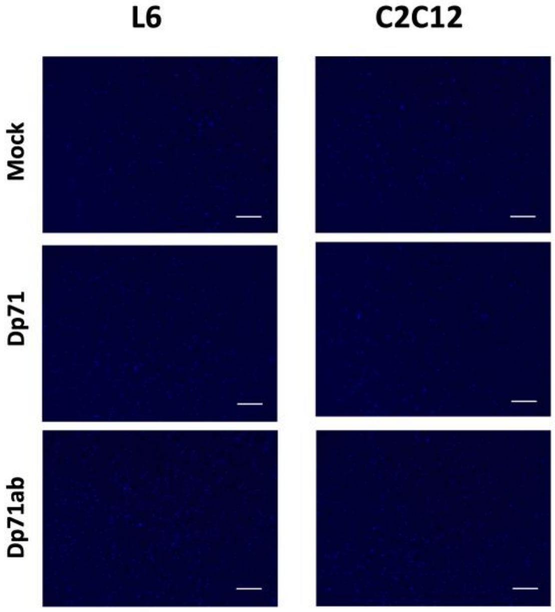

Supplementary Figure 1. Rat and mouse myoblasts cultured for 72 hrs in growth medium.

Representative images of rat (L6) and mouse (C2C12) myoblasts at 72 hrs of the culture are shown. Cell number of L6 and C2C12 cells was counted after Hoechst staining using a fluorescence microscopy. Images obtained from cells transfected with Mock, Dp71 and Dp71ab plasmids are shown at top, middle and bottom columns, respectively.

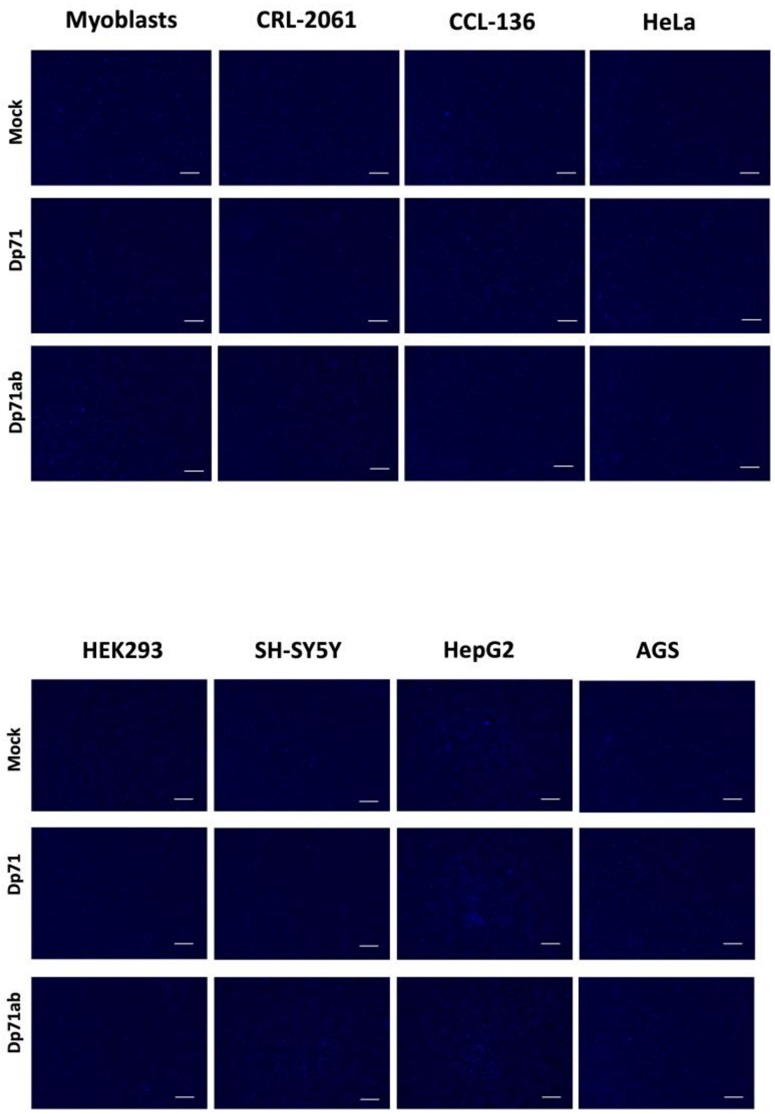

Supplementary Figure 2. Human cell lines cultured for 72 hrs in growth medium. Seven human cell lines including Myoblast, CRL-2061, CCL-136, HeLa, HEK293, SH-SY5Y, HepG2 and AGS cells were cultured for 72 hrs in the growth medium and their cell numbers were counted after Hoechst staining using a fluorescence microscopy. Images obtained from cells transfected with Mock, Dp71 and Dp71ab plasmids are shown at top, middle and bottom columns, respectively.
